# The first crystal structure of a DNA-free nuclear receptor DNA binding domain sheds light on DNA-driven allostery in the glucocorticoid receptor

**DOI:** 10.1038/s41598-018-31812-9

**Published:** 2018-09-10

**Authors:** Filipp Frank, C. Denise Okafor, Eric A. Ortlund

**Affiliations:** Department of Biochemistry, Emory University School of Medicine, Atlanta, GA 30322 USA

## Abstract

The glucocorticoid receptor (GR) is a steroid hormone receptor of the nuclear receptor family that regulates gene expression in response to glucocorticoid hormone signaling. Interaction with specific GR DNA binding sequences causes conformational changes in the GR DNA binding domain (DBD) that result in recruitment of specific sets of co-regulators that determine transcriptional outcomes. We have solved the crystal structure of GR DBD in its DNA-free state, the first such crystal structure from any nuclear receptor. In contrast to previous NMR structures, this crystal structure reveals that free GR DBD adopts a conformation very similar to DNA-bound states. The lever arm region is the most variable element in the free GR DBD. Molecular dynamics of the free GR DBD as well as GR DBD bound to activating and repressive DNA elements confirm lever arm flexibility in all functional states. Cluster analysis of lever arm conformations during simulations shows that DNA binding and dimerization cause a reduction in the number of conformations sampled by the lever arm. These results reveal that DNA binding and dimerization drive conformational selection in the GR DBD lever arm region and show how DNA allosterically controls GR structure and dynamics.

## Introduction

Glucocorticoids (GCs) are steroid hormones that are involved in various fundamental processes at the cellular and organismal level. Their anti-inflammatory and immune-suppressive functions have made them one of the most widely used class of drugs. However, their additional, undesired metabolic activities can cause severe side effects and limit their therapeutic use^1,2^. Separating beneficial anti-inflammatory from adverse side effects is the main challenge for improving GC drug performance.

In cells, GCs bind to the glucocorticoid receptor (GR), a steroid hormone nuclear receptor that regulates the expression of thousands of genes^3^. GR integrates hormone signals in a cell-type and gene-specific manner to enhance or repress transcriptional activity. GC binding to the GR ligand binding domain (LBD) in the cytoplasm causes it to translocate to the nucleus where its DNA binding domain (DBD) recognizes and binds to specific GR DNA binding sequences (GBSs) or inverted repeat GBSs (IR-GBSs). A GBS or IR-GBS can modulate expression of nearby genes by transactivation or transrepression, respectively^4–6^. The consensus GBS is ~15 base pairs in length and consists of two hexameric half-sites, AGAACA, separated by a spacer of three base pairs^7^. This spacer length is strictly required for dimerization of two GR DBDs in a head-to-head orientation with their dimerization loops (D-loops) facing and contacting each other^8^. This dimerization mode facilitates highly cooperative DNA binding^9^. The consensus IR-GBS, CTCC(N)_0–2_GGAGA, does not require a strict spacing between its two binding sites^6^ and two GR molecules interact with IR-GBSs in a head-to-tail orientation on opposite sides of the DNA with their D-loops directed away from each other^5^. GR – IR-GBS interaction is characterized by negative cooperativity with one high affinity site and a second site of much lower affinity^5^.

Transactivation and transrepression require the selective recruitment of co-activators and co-repressors, respectively^10–13^. Furthermore, the level of transactivation itself is strongly affected by the exact sequence of the GBS^4^. These observations suggest that differences in DNA modulate GR structure, which is then sensed by its coregulators. In fact, crystal structures of GR DBD bound to various GBSs revealed a loop region in the DBD, the lever arm between the DNA recognition helix and the dimerization loop, whose conformation is sensitive to the bound DNA’s sequence^4^. Different GBSs are found with different conformations of the lever arm region and mediate different transcriptional responses^4^. Similarly, DNA sequence also modulates the conformation of the dimerization loop in a head-to-head dimer^14^. Therefore, DNA may allosterically affect GR structure and, as a consequence, transcription^1^.

GRɣ, a GR isoform resulting from alternative splicing, differs from GRα only by a single arginine inserted into the lever arm. This difference results in distinct DNA binding properties, protein interaction partners, differential regulation of specific target genes and distinct functional roles at cellular level^4,15–17^. Together these studies suggest that the lever arm region is a flexible element whose specific conformational and dynamical state in a particular GR-DNA complex is responsible for the selective recruitment of co-factors to specific genomic loci.

While there are numerous crystal structures of GR DBD in complex with DNA of varying sequences, the only available structures of GR DBD in its free state were solved using NMR^18–20^. Comparison of free DBD NMR structures with crystal structures of the DNA-bound form suggested that DNA-binding is accompanied by specific conformational changes involving mostly the C-terminal half of the molecule^18–20^. In particular, no evidence was observed for the short distorted helix that forms part of the second zinc finger binding domain^20^. The authors reasoned that formation of this helix upon DNA binding may give rise to a conformational change in the adjacent D-loop, thus facilitating cooperative binding. Further, large conformational changes compared to the DNA-bound state were observed in the lever arm region^19^. It was suggested that rotation of the protein backbone around a point in the lever arm acts as a conformational relay through which small changes in the DNA recognition helix result in a reorientation of the D-loop.

Here, we have solved the crystal structure of the GR DBD in its DNA-free state. Its structure is similar to the DNA-bound form with differences in the lever arm and D-loop that are not as pronounced as previously observed in NMR structures. Molecular dynamics simulations of GR-DBD in complex with a GBS, an IR-GBS, and the free state provide evidence that DNA binding and dimerization reduce conformational sampling of the lever arm region in a sequence-specific manner. These observations offer a mechanistic explanation for allosteric control of GR structure and its transcriptional output by specific DNA sequences.

## Results

### General description of the crystal structure

**Figure 1:**
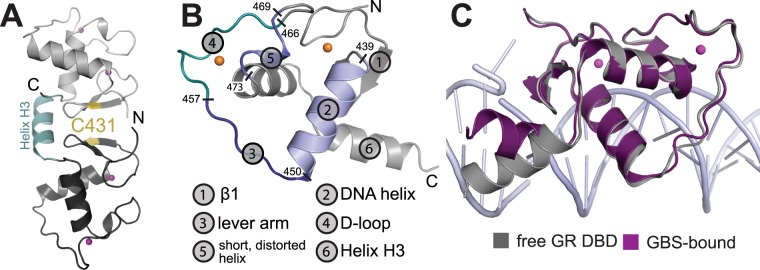
Crystal structure of the DNA-free GR DBD. **(A)** Dimerization via formation of a disulfide between adjacent C431 residues. **(B)** Overall structure of the free GR-DBD with important structural elements highlighted. **(C)** The orientation of helix H3 differs between the free DBD and the DNA-bound state. In the DNA-bound state H3 lies across the major groove and makes interactions with the DNA phosphate backbone. The conformation of H3 found in the free state is rotated by ~60 degrees so that the helix lines up perpendicular to the major groove.

The GR DBD crystallized in its free form in space group P1 with eight molecules in the asymmetric unit (Table 1). The eight molecules contain four dimers that are linked by a disulfide bridge via Cys431 (Fig. 1A). While this dimer appears to be advantageous for crystallization (no reducing agents were present during crystallization) it is not likely to form under physiological conditions in the intracellular reducing environment. Purified GR DBD is monomeric in solution^21^.

**Table 1:** X-ray diffraction data.

Wavelength	1.000
Resolution range	36.04–2.5 (2.589–2.5)
Space group	P 1
Unit cell	65.8, 65.4, 72.7; 71.3, 85.0, 68.4
Unique reflections	33141 (2095)
Multiplicity	2.4 (1.4)
Completeness (%)	89.47 (56.85)
Mean I/sigma(I)	14.5 (1.3)
Wilson B-factor	53.58
R_meas_	0.18 (0.65)
R_pim_	0.10 (0.46)
CC_1/2_	(0.56)
Reflections used in refinement	33024 (2092)
Reflections used for R-free	1991 (126)
R-work	0.22 (0.33)
R-free	0.22 (0.35)
Number of non-hydrogen atoms	4707
macromolecules	4664
Zn^2+^	16
solvent	27
Protein residues	619
RMS(bonds)	0.006
RMS(angles)	1.00
Ramachandran favored (%)	97.1
Ramachandran allowed (%)	2.6
Ramachandran outliers (%)	0.3
Rotamer outliers (%)	0.59
Clashscore	10
Average B-factor	76.78
macromolecules	76.96
ligands	54.02
solvent	58.69

Statistics for the highest-resolution shell are shown in parentheses.

Within each dimer, one molecule shows clear density within the C-terminal helix (H3) following the core DBD, whereas this stretch is mostly disordered in the other molecule (Fig. 1A,B). H3 had been resolved to varying extents in previous DNA-bound crystal structures. It lines up over the minor groove, makes contact with the DNA backbone 3 bp upstream of the GBS, and contains five lysine residues for potential further DNA interactions^4^. In the full-length receptor, the DBD is followed by a short linker connecting directly to the LBD. The orientation of H3 along the minor groove may thus serve to constrain the LBD to a conformation competent for dimerization. In the free state observed here, H3 is rotated by approximately 60 degrees so that it would lie across the minor groove of a bound DNA (Fig. 1C). This conformation is likely influenced by crystallization and the formation of the disulfide-bridged dimer. However, the current structure suggests that the orientation of H3 relative to the core DBD is not fixed in the absence of DNA and adopts its active conformation only upon GR binding to a recognition sequence.

**Figure 2:**
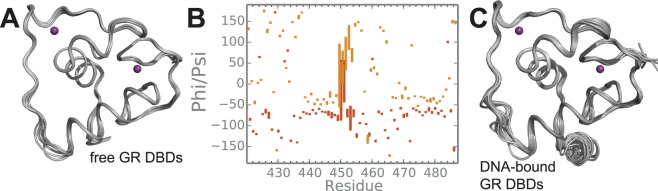
Lever arm flexibility. **(A)** Structural alignment of the eight monomers found in the asymmetric unit of the crystal. **(B)** Box plot of the Phi and Psi backbone dihedral angles observed in the eight GR DBD monomers. Each box represents the interquartile range. The highest variability is observed in the lever arm region. **(C)** Structural alignment of crystal structures of the GR DBD bound to different GBSs.

**Figure 3:**
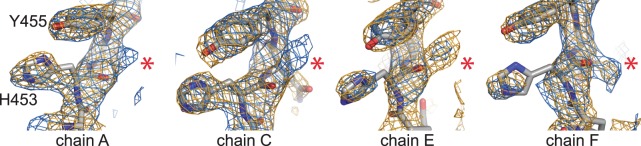
Electron density indicative of multiple conformations for His453. Shown in orange is a 2F_o_-F_c_ map at 1.5σ and in blue is a composite omit map contoured at 2σ. The red asterisks mark the alternate location of the His453 side chain in the “in” conformation.

The core DBD (residues 421–491) of the individual molecules within the unit cell are very similar in structure (Fig. 2A; average pairwise RMSD = 0.49 Å). The largest differences are located within the lever arm region (Fig. 2A,B) and are similar in extent to the variations observed in GR DBD bound to different GBSs (Fig. 2C)^3,4^. His453 is known to either pack into the protein’s core or flip outwards depending on the sequence of bound DNA^4^. In the free crystal structure, His453 is flipped out in all molecules. However, close inspection of electron density shows evidence for an alternative, packed conformation in four molecules (Fig. 3). These observations suggest that the lever arm, including His453, is mobile in the free state and samples multiple conformations resembling the various structures in the presence of different DNA sequences.

### Significant differences with previous free DBD structures solved by NMR

Previous structures of free GR DBD, solved by NMR, reveal significant conformational changes compared to the DNA-bound form found in crystal structures^18–20^. Hydrogen bonding interactions defining the short antiparallel beta-sheet in the N-terminal Zn finger were not present in NMR structures^8,21^. The short, distorted helix in the second zinc finger (residues 469–473) had not been unambiguously observed in NMR experiments^18,20^. The largest change in conformation was described in the adjacent D-loop (residues 457–465), which mediates dimerization of GR DBD on DNA^18^. Finally, NMR studies identified the lever arm region (residues 450–456) as a hinge around which small differences within the preceding DNA reading helix are amplified to cause large conformational changes in the succeeding D-loop^19^. Although some of the conformational differences in NMR structures may have been due to a lack of well-defined NMR constraints^18^, it had been accepted that DNA binding is accompanied by significant conformational changes in the DBD.

**Figure 4:**
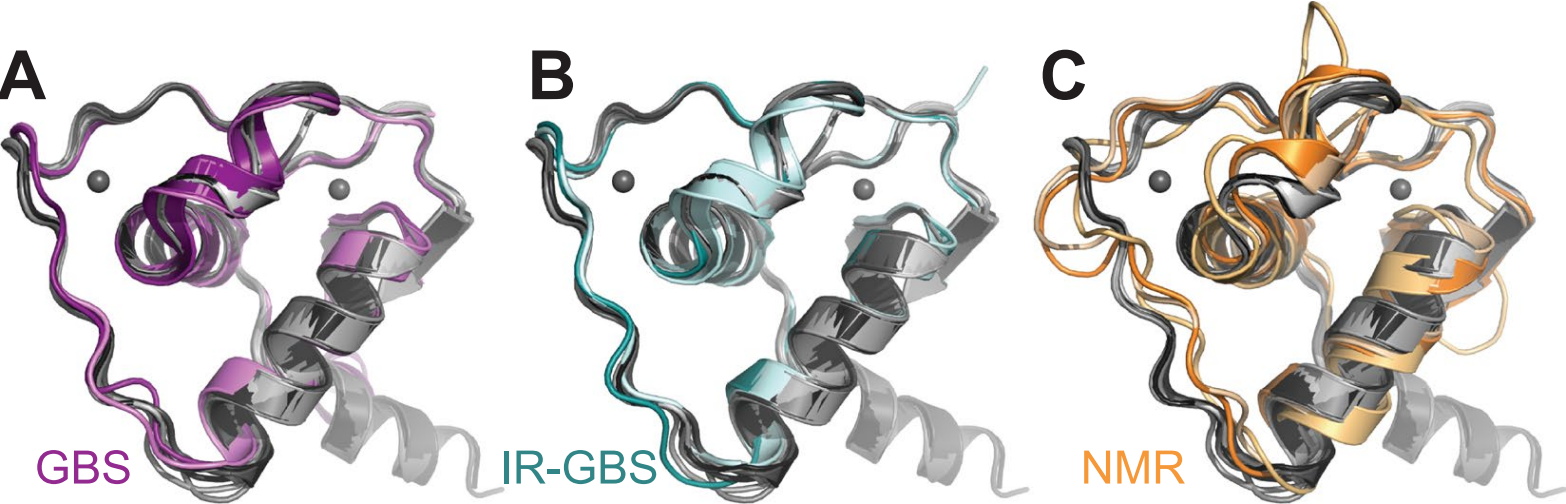
Structural alignment of DNA-free GR DBD with previous structures. **(A–C)** Structural alignments of the 8 monomers found in the crystal structures of free GR-DBD (in grey) overlaid with structures of DNA-bound GR DBD from crystal structures 3G99 (**A**) and 4HN5 (**B**) and DNA-free GR DBD from NMR structures 1GDC, 1RGD, and 2GDA (**C**).

**Figure 5:**
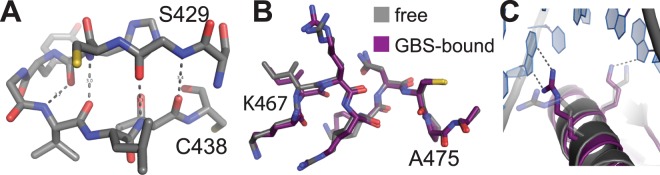
Structural elements of GR DBD in the DNA-free state. **(A)**. Hydrogen-bonds forming the antiparallel beta-sheet in the N-terminal Zn finger in the DNA-free GR DBD structure. (**B)** The short, distorted helix in the C-terminal Zn finger in DNA-bound (PDB ID: 3G99) and free GR DBD adopt the same conformation. **(C)** Conformation of the DNA reading helix. Shown is a structural alignment of a representative molecule of DNA-free GR DBD with DNA-bound GR-DBD (3G99). The DNA reading helix aligns well between the two proteins and the position of two residues that make direct contact with the DNA in 3G99 is unchanged in the DNA-free structure.

Comparison of the current structure of free GR DBD with NMR structures as well as DNA-bound crystal structures shows that the free GR DBD crystal structure described here is more similar to the DNA-bound form (Fig. 4A and B; RMSDs 0.72–1.08 Å; Table 2) than to the solution structures of free GR DBD (Fig. 4C; RMSDs 1.83–2.77 Å; Table 2). In particular, the N-terminal beta-sheet as well as the short, distorted helix are formed and identical in structure to the DNA-bound state (Fig. 5A,B) and the D-loop conformation is similar to the one found in DNA-bound structures (Fig. 4A,B). The conformation around the lever arm region is characterized by a certain degree of flexibility in the current structures as evidenced by multiple different conformations in the eight monomers of the unit cell as well as electron density indicative of multiple distinct conformations in His453 within the monomers (Fig. 3). However, it is very different from the conformations found in NMR structures and more closely resembles the DNA-bound state (Fig. 4).

**Table 2:** Pairwise RMSD between different GR DBD structures.

*RMSD [Å]*	1GDC	1RGD	2GDA	3G99_A	3G99_B	4HN5_A	4HN5_B
Free GR DBD	2.03	2.77	1.83	0.976	1.08	0.92	0.73
1GDC		2.69	0.759	1.95	1.91	1.94	1.99
1RGD			2.63	2.79	2.92	2.76	2.73
2GDA				1.80	1.80	1.80	1.78
3G99_A					0.614	0.67	0.54
3G99_B						0.67	0.62
4HN5_A							0.70

For the free GR DBD, molecule C of the crystal structure described here was used.

Changes between the DNA-free and DNA-bound crystal structures are minor. Differences are located around the mobile lever arm region and extend into the D-loop (Fig. 4A,B). Interestingly, the peptide backbone conformation of the DNA-binding helix, including the residues in direct contact with DNA, is essentially unchanged between the free and GBS DNA-bound forms (Fig. 5C). However, the side chains of these residues are solvent-exposed in the free form and as a result they are highly dynamic, and their positions are not resolved. Thus, the side chains appear to become ordered into their active conformations only upon contact with DNA.

An important advantage of NMR spectroscopy over crystallography is that it probes macromolecular structure in solution, rather than in a solid, crystalline state. Under these conditions dynamic protein elements fluctuate between different conformations as they would in their physiological environment in the cell. The fact that no single local conformation for such elements exists in solution may result in poorly defined regions. In a crystalline environment, on the other hand, the protein is generally stabilized in a single, low energy state. In the asymmetric portion of the unit cell, we observe eight independent DNA-free GR DBD molecules, which closely resembles the DNA-bound state observed in other crystal structures. This suggests that the DNA binding conformation is accessible to the GR DBD in solution

### The lever arm is flexible in MD simulations

Lever arm conformation is sensitive to the presence or absence of DNA, the sequence of bound DNA in a GR-DNA complex, and even to external factors such as buffer conditions during crystallization^4^. One flexible part of the lever arm is residue His453, which can adopt two distinct conformations: packed in the core of the protein (“in”) or flipped outwards (“out”). Together, these observations suggest that dynamics in this region play an important role in GR DBD function. To explore this further, we carried out two replicates each of 1.0 µs molecular dynamics simulations containing GR DBD molecules in different functional states: (i) the free GR DBD reported here (molecule C of the crystal structure), (ii) a GR DBD dimer bound to a representative GBS containing a perfect palindromic hexameric repeat with two high-affinity binding sites (GBS1 and GBS2; PDB 3G99, chains A and B, respectively)^4^, and a GR DBD dimer bound to a palindromic IR-GBS (PDB 4HN5) containing a high-affinity (IR-GBS1) and a low-affinity (IR-GBS2) binding site^5^. The two GR binding sites within the IR-GBS are characterized by strong negative cooperativity of binding^5^ and IR-GBS2 is likely not fully occupied *in vivo*. Its presence in the structure resulted from stabilizing contacts that facilitated crystallization.

To further validate results from this initial set of simulations, two alternative systems for the DNA-free state and the GBS bound state were simulated, also in duplicate: (i) molecule A of the crystal structure reported here and (ii) PDB ID 3G9J^4^, a structure similar to 3G99, but with 18 nucleotide DNA strands instead of 16.

**Figure 6:**
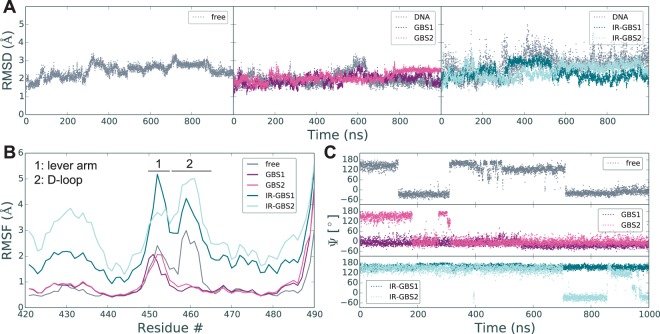
Overview of MD simulations. **(A)** C_α_ root mean square displacements (RMSDs) of replicate #1 simulations. **(B)** C_α_ root mean square fluctuations (RMSFs) of replicate #1 simulations. **(C)** Dynamics of His453 flipping between ‘in’ and ‘out’ conformations. Shown are the trajectories of the His453 *psi* dihedral angle, which reflects how the residue flips between its ‘in’ (~−28°) and ‘out’ (~143°) conformation.

All GR DBD molecules were characterized by stable root mean square displacements (RMSDs) over the course of the entire simulation (Fig. 6A and Supplementary Figs 1 and 2). Root mean square fluctuations (RMSFs), a measure of flexibility, reveal the lever arm and D-loop as the most mobile regions in the proteins (Fig. 6B and Supplementary Figs 3 and 4). As expected, the head-to-head dimerization between GBS1 and GBS2 stabilized the D-loop of those molecules, which resulted in lower RMSFs in that region compared to the free state or the IR-GBS1 and IR-GBS2 states. Due to their spatial proximity, stabilization of the D-loop may have direct effects on the dynamical behavior of the lever arm region.

Interestingly, the IR-GBS complex exhibited the greatest RMSFs amongst all the simulations, in both replicates. This was surprising since the free structure and the GBS complex behaved similarly. It appears that binding to an IR-GBS sequence induces significant flexibility in the DBD compared to the free or GBS-bound state. This is likely due to the strong negative cooperativity which disfavors simultaneous binding of two GR molecules at this DNA sequence. Previous studies showed that strong allosteric communication flows through the DNA to mediate this effect.

His453 in the lever arm was observed to flip between its two main conformations in a number of cases (Fig. 6C). The conformation of His453 is found in two main states represented by *psi* backbone dihedral angles of 143° +/− 20° (out) and −28° +/− 11° (in), respectively. Switching between these states is rapid with a potential on-pathway intermediate at 119° +/− 13° in the out-to-in transition. These data underscore the dynamic behavior of this residue and are consistent with the multiple conformations detected in crystal structures.

### Molecular dynamics uncover distinct dynamic properties for different functional states of the GR DBD

**Figure 7:**
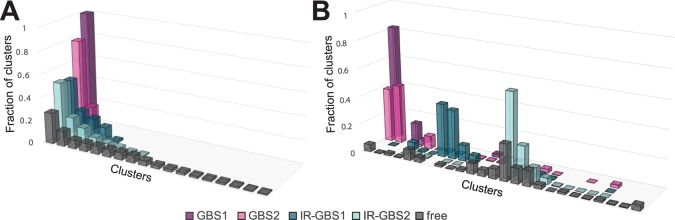
Cluster analysis for lever arm conformations. **(A)** Clustering of individual MD trajectories shows trends in the conformational space sampled in different states of GR. For each molecule clusters are ordered by decreasing frequency. **(B)** Combined clustering of the duplicate trajectories for DNA-free GR DBD (molecule **C**), 4HN5, and 3G99 shows which clusters are shared between the different molecules. The results show that the two monomers bound to a GBS spend ~40% of their time in the same conformation. Further significant overlap is observed between free GR and the low affinity binding site within the IR-GBS complex (IR-GBS2). Molecule IR-GBS1 does not have significant overlap with any other molecules.

Previous MD simulations over 50–100 ns of GR DBD in different states revealed small changes in lever arm RMSF values that correlated with transcriptional output of the simulated complexes^22,23^. Considering all of the 1µs simulations performed in this study, we did not observe significant, systematic differences in lever arm RMSFs between the DNA-free and the DNA-bound state. To more closely examine the relationship between lever arm dynamical behavior and GR DBD functional state, we performed a conformational clustering analysis. For each simulation, 50,000 frames, equally distributed in time over the course of the trajectories, were clustered based on their lever arm conformations. Clustering frames from each trajectory independently showed a trend in the number of conformations sampled by each monomer. GBS1 molecules maintained a single conformation over the entire simulation, whereas GBS2 is distributed over two and three separate clusters in the duplicate simulations (Fig. 7A and Supplementary Fig. 5). IR-GBS1 and IR-GBS2 conformations distributed into an intermediate number of clusters (IR-GBS1: 5 and 6; IR-GBS2: 8 and 3). Strikingly, the greatest number of clusters was identified for simulations of the DNA-free state: 12 and 18 for molecule C, and 10 and 8 for molecule A (Fig. 7A and Supplementary Fig. 5). The increase in the number of clusters in the free structure was not a consequence of a general increase in protein mobility as RMSF values were comparable to simulations of GBS1 and GBS2. It more likely results from the lack of stabilizing contacts in the adjacent D-loop that are present in the GBS structures. The resulting increase in D-loop flexibility may amplify the conformational space accessible to the lever arm. D-loop flexibility alone, however, does not explain the entire effect on lever arm clustering since IR-GBS structures would be expected to sample more distinct conformations. The smaller number of clusters for IR-GBS1 and IR-GBS2 may be explained by stabilization through neighboring contacts with DNA. Thus, the clustering analysis suggests that, while flexible to a similar degree in all states, lever arm conformational sampling is progressively reduced with the addition of nearby contacts through DNA binding via the DNA-helix and dimerization via the D-loop.

The clustering analysis was also carried out on the two alternative systems that were simulated for the DNA-free state and the GBS bound state. Again, the DNA-free state was characterized by a greater number of clusters compared to the DNA-bound states, confirming that these observations are robust in different structural contexts (Supplementary Fig. 6).

To more directly compare lever arm conformations across the different simulations, we performed a combined cluster analysis using frames equally distributed over the duplicate simulations of the initial set of structures (GBS1 and GBS2, IR-GBS1, and IR-GBS2, and DNA free; Fig. 7B). The analysis identified a total of 25 unique clusters among the trajectories. The results of the two clustering analyses are in overall agreement in that lever arm conformations identified for GBS1 and GBS2 were distributed over a smaller number of clusters compared to IR-GBS1 and IR-GBS2. The free state was most heterogeneous. Examination of the degree of conformational overlap between the different DBD monomers reveals that the greatest overlap existed between GBS1 and GBS2 (~40%; Fig. 7B). Another significant overlap occurred between the free and IR-GBS2 states, which share three clusters covering at least 10% of the time each. Interestingly, IR-GBS1 is somewhat separate from the other states having only minor overlap with other molecules and spending most of its time in conformations exclusive to this state (Fig. 7B).

The results of the clustering analyses allow several conclusions. First, the large number of clusters observed in the free state shows that the lever arm samples multiple distinctive conformations in the absence of DNA. Conformational sampling is restricted upon binding to DNA and is further reduced by dimerization via the D-loop as seen in complexes with a GBS. This suggests that DNA binding and dimerization allosterically bias the conformation of the lever arm region in a specific DBD-DNA complex. Importantly, this mechanism is consistent with changes in GR DBD chemical shifts observed by ^1^H-^15^N-HSQC experiments in the presence and absence of DNA^14^. Second, while conformational sampling of the lever arm is reduced in DNA-bound complexes, it retains enough flexibility to sample a small number of distinct conformations. These may provide the ability to interact with different co-regulators while bound to a particular DNA sequence and the recruitment of a specific binding partner may depend on further signals. The different regulatory outcomes achieved by GBS and IR-GBS sequences may be explained by a combination of distinct lever arm conformations and increased lever arm conformational sampling in GR bound to an IR-GBS.

## Conclusions

We have solved the first crystal structure of a free steroid receptor DNA binding domain. The structure contains eight independent molecules in the asymmetric unit and superposition of these molecules reveals that the free GR DBD is very similar to its DNA-bound form. Differences are minor and located mostly within the lever arm and the adjacent D-loop. This is in contrast to NMR studies of the free GR DBD, which had identified a number of protein segments with major conformational differences compared to DNA-bound crystal structures^18–20^. The absence of any large conformational changes between the DNA-free and DNA-bound crystal structures suggests that minor structural variations found within the lever arm and the D-loop are responsible for their differential functional properties. This view is consistent with previous studies showing that the lever arm and D-loop are the main sites of variability in structures of GR DBD bound to DNA of different sequences^4,14,23^.

In addition to variability between different GR DBD crystal structures, the lever arm is also the most flexible element in molecular dynamics simulations, consistent with previous reports^22,23^. We reasoned that differences in its intrinsic dynamics are responsible for functional differences. However, lever arm RMSF values did not display significant differences between the DNA-free and DNA-bound states. Instead, a clustering analysis of the MD trajectories based on lever arm conformation revealed differences between the different functional states: GBS-bound molecules spent most of their trajectories in the same conformational cluster, IR-GBS-bound molecules were characterized by an intermediate number of clusters, and the lever arm of DNA-free GR DBD sampled the most diverse conformations (Fig. 7A). A combined cluster analysis further uncovered that the two GBS-bound molecules populated a shared cluster, while there was only minimal overlap with clusters populated in any of the other simulated molecules. Thus, clustering analysis of MD trajectories is able to distinguish the different functional states of the GR DBD.

Together, the data presented here show that large-scale conformational changes in the GR DBD may not be required for DNA binding. It is rather more likely that the lever arm undergoes conformational selection from a highly heterogeneous collection of clusters in the free state to a small number of unique clusters in the functional, DNA-bound state. Furthermore, lever arm clusters of DNA-bound GR DBD correlated with the direction of transcriptional output mediated by the particular DNA segments. These insights contribute to our understanding of how DNA sequence inputs control the conformational landscape of the GR DBD, which may have consequences for the transcriptional regulation of target genes.

## Methods

### Protein expression and purification

The GR DBD (residues 418–517) was cloned into pSmt3 vector for expression with an N-terminal His6-Sumo-tag^24^ using BamHI and NotI restriction sites. The protein was expressed in E coli BL21 DE3. Cells were grown to an OD_600_ of 0.8–1.0 and overexpression was induced with 0.5 mM IPTG for 4 hours at 37 C. Protein was purified by nickel affinity chromatography, followed by cleavage of the His_6_-Sumo-tag using Ulp-1 enzyme. After removal of the His6-Sumo-tag by a second round of nickel-affinity chromatography, the protein was concentrated and purified by size exclusion chromatography in 25 mM Tris pH7.5, 100 mM NaCl, and 5% glycerol. Purified protein was flash frozen and stored at a concentration of ~5 µg/µL.

### Protein crystallization and data collection

In an attempt to crystallize GR DBD in complex with an RNA GBS mimic present in the lncRNA Gas5^25^ protein was mixed with a 35nt short RNA hairpin (sequence: GCTCCCAGTGGTCTTTGTAGACTGCCTGATGGAGC) at a 1.1:1 ratio of protein to RNA at a concentration of ~4 µg/µL. Protein was crystallized by hanging drop vapor diffusion at 18 °C in solutions containing 25% PEG3350, 50 mM Tris pH8.5, and 200 mM di-sodium tartrate. Crystals were cryo-protected by addition of well solution containing 20% glycerol and flash frozen in liquid nitrogen. Data were collected remotely from the South East Regional Collaborative Access Team (SER-CAT) at the Advanced Photon Source 22ID beamline (Argonne National Laboratories, Chicago). Data were processed and scaled using *HKL2000* (ref Ottwinowski) and phased by molecular replacement using Phaser-MR in *PHENIX*^26^. PDB 4HN5 chain A was used as the search model. Model building and refinement were carried out using *Coot*^27^ and *PHENIX*^26^. No electron density indicating the presence of nucleic acid was found anywhere in the unit cell.

### Model construction for molecular dynamics simulations

Three GR DBD complexes were prepared for molecular dynamics simulations: 1) Free GR DBD (PDB 6CFN) (GR monomer), 2) GR-GBS (PDB 3G99) (dimer), and 3) GR-IR-GBS (PDB 4HN5) (two monomers). For consistency, all GR sequences were truncated to contain residues 421–490 (human numbering).

### Molecular dynamics simulations

The complexes were solvated in an octahedral box of TIP3P water with a 10 Å buffer around the protein/protein-DNA complexes. Na^+^ and Cl^-^ ions were added to neutralize the protein and achieve physiological conditions. All systems were set up using xleap in *AmberTools* (version 17) and force fields contained in Amber16^28^. The *ff14SB* force field was used for protein and OL15 for DNA. Zinc metal centers were modeled with the zinc Amber force field (ZAFF)^29^. All minimizations and simulations were performed with *Amber16*. Systems were minimized with 5000 steps of steepest descent followed by 5000 steps of conjugate gradient minimization with 500-kcal/mol∙Å^2^ restraints on all non-solvent atoms. The minimization was repeated sequentially with the following conditions: i) 500-kcal/mol∙Å^2^ restraints on protein and Zn atoms only (for GR-DNA complexes), ii) 500-kcal/mol∙Å^2^ restraints on Zn^2+^ atoms and coordinating cysteines only, iii) 500-kcal/mol∙Å^2^ restraints on Zn atoms only, and iv) no restraints. The systems were heated from 0 to 300 K using a 100-ps run with constant volume periodic boundaries and 10-kcal/mol∙Å^2^ restraints on all non-solvent atoms. The following stepwise equilibration was performed using the NPT ensemble: i) 1 ns with 10-kcal/mol∙Å^2^ on all non-solvent atoms; ii) 1 ns with 10-kcal/mol∙Å^2^ on protein and Zn atoms only (GR-DNA complexes), iii) 1 ns with 10-kcal/mol∙Å^2^ on Zn^2+^ atoms and coordinating cysteines only; and iv) 1 ns with 10-kcal/mol∙Å^2^ on Zn atoms only. All restraints were removed and 1 µs production simulations were performed for each system in the NPT ensemble. A 2-fs time step was used and all bonds between heavy atoms and hydrogens were fixed with the SHAKE algorithm^30^. A cutoff distance of 10 Å was used to evaluate long-range electrostatics with particle mesh Ewald and for van der Waals forces. For analysis, 50,000 evenly spaced frames were obtained from each simulation.

### Analysis

Structural averaging and analysis were performed with the *CPPTRAJ* module^31^ of *AmberTools*. The MMTSB toolset^32^ was used to perform a cluster analysis of GR monomer conformations. The *k-means* clustering algorithm (kclust) was used with a 2.3 Å RMSD cutoff radius. This radius was selected as the optimal radius to reflect the conformational variability of the selected fragment (i.e. the lever arm) across all GR monomers. 50,000 evenly spaced snapshots from each simulation were RMSD fitted to the lever arm and clustered. To identify sampled conformations that overlap between the free and DNA-bound GR monomers in the two replicate simulations, 5,000 evenly spaced snapshots from each monomer (50,000 total) were combined, RMSD fitted and clustered.

## Electronic supplementary material

Supplementary Information
