# Supplementary material for: The first crystal structure of a DNA-free nuclear receptor DNA binding domain sheds light on DNA-driven allostery in the glucocorticoid receptor
Source: Sci Rep. 2018 Sep 10;8:13497. doi: 10.1038/s41598-018-31812-9 (PMC6131172; doi:10.1038/s41598-018-31812-9)
Supplement: Supplementary file 1 — Supplementary Information [file 41598_2018_31812_MOESM1_ESM.pdf]

Supplementary Material

Title: "The first crystal structure of a DNA-free nuclear receptor DNA binding domain sheds light on DNA-driven allostery in the glucocorticoid receptor"

Authors: Filipp Frank[1], C. Denise Okafor[1], Eric A. Ortlund[1,*]

[1]Department of Biochemistry, Emory University School of Medicine, Atlanta, GA 30322, USA

[*]Corresponding author:

Eric A. Ortlund,

eortlund@emory.edu,

Emory University,

Department of Biochemistry,

1510 Clifton Rd. NE, Atlanta, GA, 30322, USA.

Supplementary Figure 1:

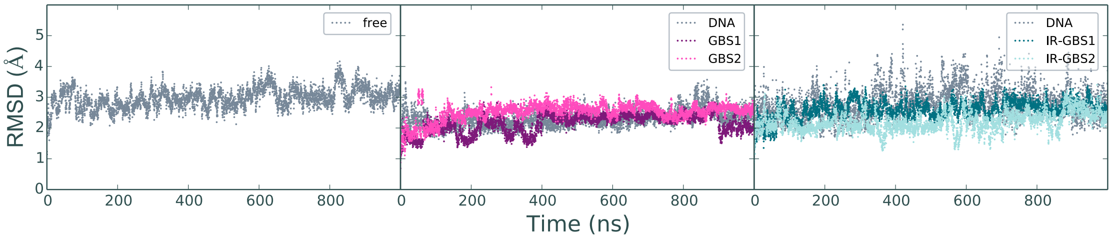

Supplementary Figure 1: C_α root mean square displacements (RMSDs) of replicate #2 simulations for DNA-free GR DBD molecule C and PDB IDs 3g99 (GBS) and 4HN5 (IR-GBS).

Supplementary Figure 2:

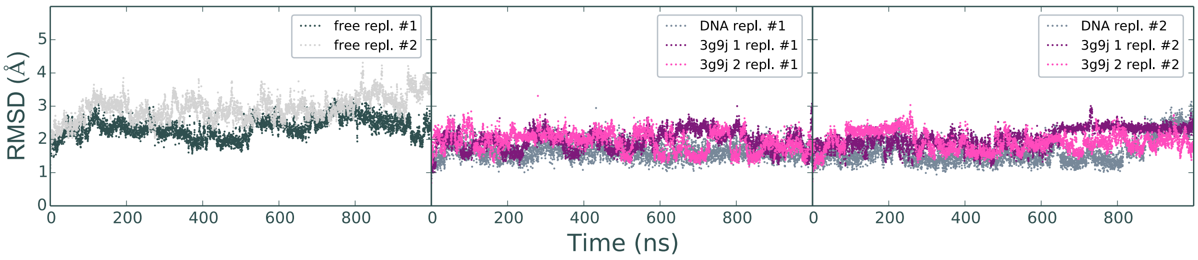

Supplementary Figure 2: C_α root mean square displacements (RMSDs) of duplicate MD simulations for DNA-free GR DBD molecule A and PDB ID 3g9j. These structures represent alternative systems equivalent to the simulations presented in the main text and serve to strengthen conclusions made from analyzing those.

Supplementary Figure 3:

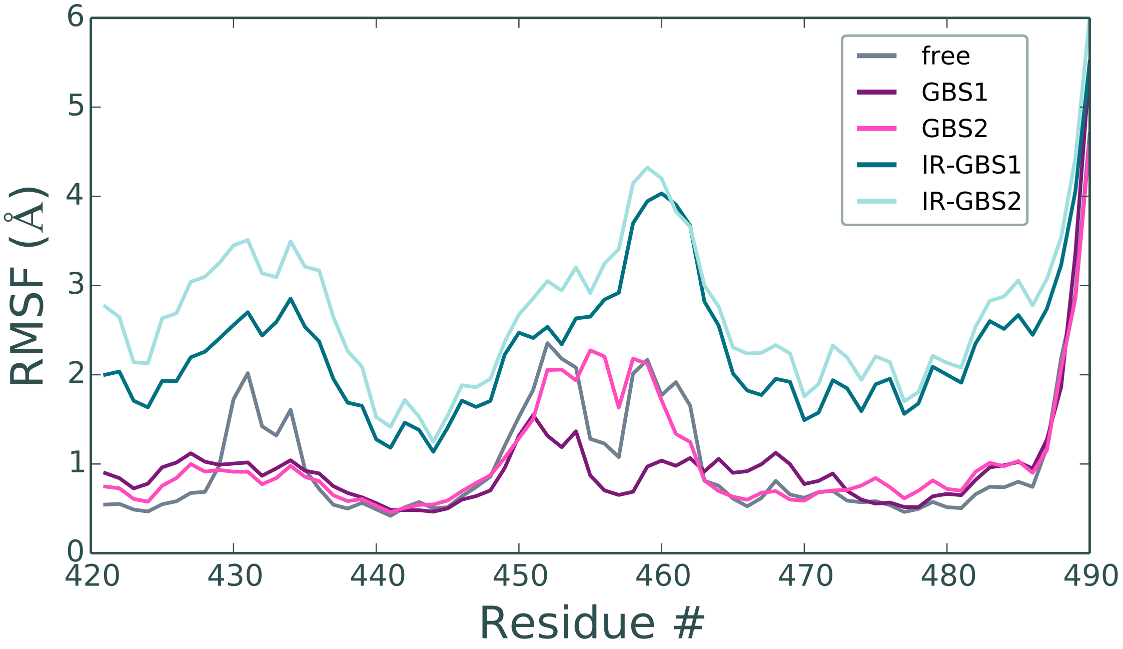

Supplementary Figure 3: C_α root mean square fluctuations (RMSFs) of replicate #2 simulations for DNA-free GR DBD molecule C and PDB IDs 3g99 (GBS) and 4HN5 (IR-GBS). Molecule GBS2 in this replicate simulation of 3g99 is the only GBS-bound monomer with increased fluctuations in the D loop (residues 457-465). Visual inspection of the simulation showed that the increase is caused by repeated breaking and reforming of the salt bridge between D462 and R460, the main contact establishing contact between adjacent D loops. This does not affect overall stability of the system as judged by stable RMSDs in both protein and DNA and suggests that the D loops retain a modest degree of flexibility even in a functional dimer.

Supplementary Figure 4:

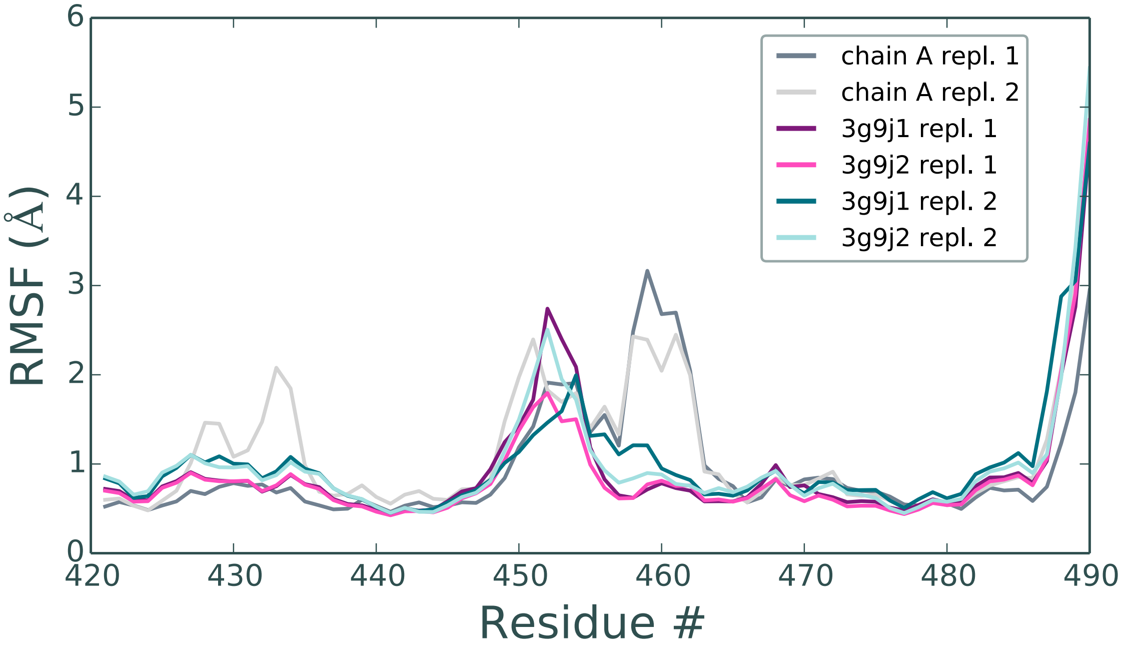

Supplementary Figure 4: C_α root mean square fluctuations (RMSFs) of duplicate MD simulations for DNA-free GR DBD molecule A and PDB ID 3g9j. These structures represent alternative systems equivalent to the simulations presented in the main text and serve to strengthen conclusions made from analyzing those.

Supplementary Figure 5:

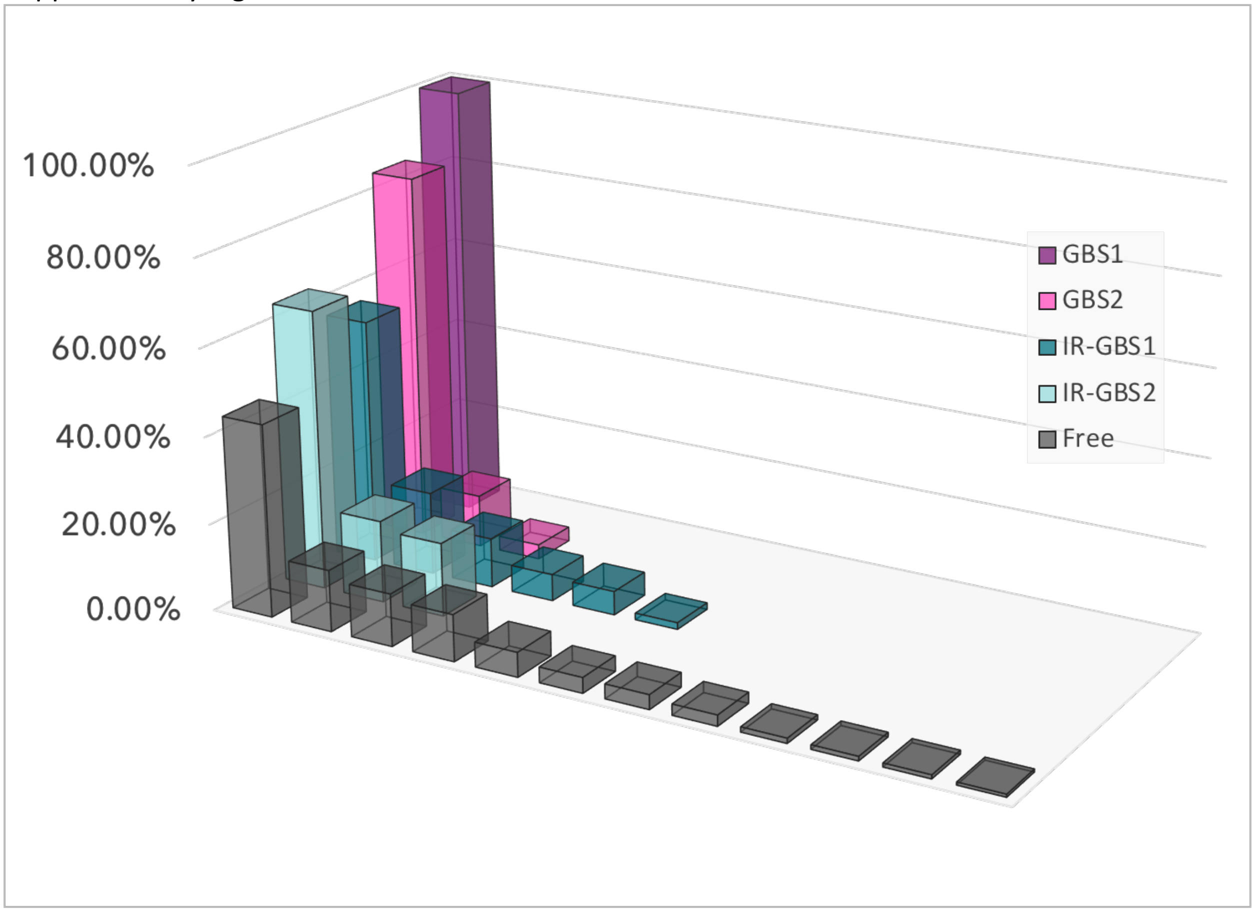

Supplementary Figure5: Cluster analysis of replicate #2 simulations for DNA-free GR DBD molecule C and PDB IDs 3g99 (GBS) and 4HN5 (IR-GBS). Lever arm conformations observed in the MD simulations were clustered and the population of clusters is reported here.

Supplementary Figure 6:

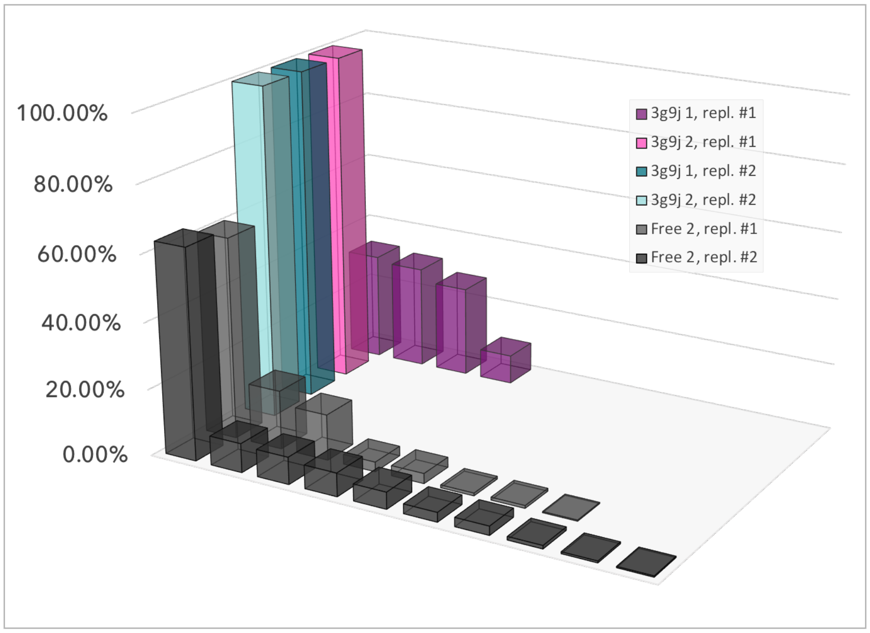

Supplementary Figure 6: Cluster analysis of alternative structures (DNA-free molecule A and PDB ID 3g9j; duplicate simulations). Lever arm conformations observed in the MD simulations were clustered and the population of clusters is reported here.
